# Evolutionary approaches for the reverse-engineering of gene regulatory networks: A study on a biologically realistic dataset

**DOI:** 10.1186/1471-2105-9-91

**Published:** 2008-02-08

**Authors:** Cédric Auliac, Vincent Frouin, Xavier Gidrol, Florence d'Alché-Buc

**Affiliations:** 1 Laboratoire Informatique, Biologie Intégrative et Systèmes Complexes (IBISC), Université d'Evry-Val d'Essonne, Evry, France; 2 Laboratoire d'Exploration Fonctionnelle des Génomes (LEFG), Institut de Radiobiologie Cellulaire et Moléculaire (IRCM), Direction des Sciences du Vivant (DSV), Commissariat à l'Énergie Atomique (CEA), Evry, France

## Abstract

**Background:**

Inferring gene regulatory networks from data requires the development of algorithms devoted to structure extraction. When only static data are available, gene interactions may be modelled by a Bayesian Network (BN) that represents the presence of direct interactions from regulators to regulees by conditional probability distributions. We used enhanced evolutionary algorithms to stochastically evolve a set of candidate BN structures and found the model that best fits data without prior knowledge.

**Results:**

We proposed various evolutionary strategies suitable for the task and tested our choices using simulated data drawn from a given bio-realistic network of 35 nodes, the so-called insulin network, which has been used in the literature for benchmarking. We assessed the inferred models against this reference to obtain statistical performance results. We then compared performances of evolutionary algorithms using two kinds of recombination operators that operate at different scales in the graphs. We introduced a niching strategy that reinforces diversity through the population and avoided trapping of the algorithm in one local minimum in the early steps of learning. We show the limited effect of the mutation operator when niching is applied. Finally, we compared our best evolutionary approach with various well known learning algorithms (MCMC, K2, greedy search, TPDA, MMHC) devoted to BN structure learning.

**Conclusion:**

We studied the behaviour of an evolutionary approach enhanced by niching for the learning of gene regulatory networks with BN. We show that this approach outperforms classical structure learning methods in elucidating the original model. These results were obtained for the learning of a bio-realistic network and, more importantly, on various small datasets. This is a suitable approach for learning transcriptional regulatory networks from real datasets without prior knowledge.

## Background

### Introduction

Biological functions result from the interaction of various macromolecules in the cell. Among the different regulatory mechanisms at work in the cell, transcriptional regulation plays an important role as it links a coding space of genes to a functional space of proteins. The availability of a wide range of genome wide experimental techniques, such as DNA microarrays or ChIP on chip, gives the modellers the opportunity to consider reverse engineering of transcriptional networks from experimental data. The elucidation of these networks is usually implemented by choosing a mathematical model to describe the interactions between a regulee and its regulators and then using the data to learn both the graph of interactions and the parameters of the mathematical model. We consider here the case when the graph structure is unknown and the learning task consists of discovering the nature of interactions.

Up to now, a variety of frameworks to infer transcriptional regulatory networks from expression profiles have been considered [1,2]. Among them, probabilistic graphical models appear to be a successful approach [3,4]. They offer an adequate representation of conditional (in)dependencies between variables and allow the management of uncertainty which is relevant in case of noisy data and stochastic processes. Regarding the learning issue, the choice between dynamic and static modelling depends mainly on data availability. Learning dynamic systems requires observations of temporal variations of gene expressions which are costly to produce while the availability of static data in complex organisms is growing. In this work, we have thus chosen to focus on learning the structure of static models using static data. Among the probabilistic models, we selected Bayesian Networks (BN) [5-7] that cover acyclic interaction networks. This class of models has often been used in the field of computational biology [3,8-13] in the past few years. Many approaches have been proposed to learn the structure of Bayesian networks [14]. Structure learning algorithms are generally based on a search within a set of candidate structures. The underlying idea is to discover the BN that best fits the available data. A scoring metric is required to assess the quality of each candidate structure with respect to the data.

To undertake the search in the huge space of BN structures [15], deterministic heuristics like greedy search [16] or the K2 algorithm [17] have been proposed. However, since the problem of structure learning is known to be NP-hard [18], stochastic heuristics like MCMC [19,20] or Evolutionary Programming [21] are usually preferred. They are supposed to overcome some limitations of deterministic search strategies, such as local optimality and dependence on the initial solution. In this work, we developed Evolutionary Algorithms (EA) to learn network structures and we applied our strategies to learn a regulatory network, called the insulin network, introduced by [22] to serve as a benchmark problem in reverse-engineering of regulatory networks. We studied various implementations of EA dedicated to BN structure optimisation and showed that EA enhanced by a niching strategy, called Deterministic Crowding, performed a pertinent exploration of the search space.

### Modelling gene regulatory networks with Bayesian networks

In this paper, gene regulatory networks are represented by (BN). Formally, BN are defined by a tuple (*G*, *P*_*G*_). *G *is a Directed Acyclic Graph (DAG) where nodes are random variables *X *= {*X*_1_,...,*X*_*n*_} and the edges encode the conditional (in)dependencies between these variables. The graph topology defines a set of parents for each node *i *: *Pa*_*i*_. Here, the random variables correspond to gene expression levels, reflecting mRNA concentrations while edges represent the interactions between parent variables (regulators) and child variables (regulee). We must emphasise that with this kind of model, no loops can be represented. This induces a strong restriction in our modelling, but several works [4,23] have shown that these approaches are successful in capturing a good part of the regulation characteristics.

According to the Markov assumption, which states that each node is independent of its non-descendants given its parents, we can factorise the joint probability distribution *P*(*X*_1_, *X*_2_, ..., *X*_*n*_) using the product of probability distributions of the *X*_*i*_'s given their parents *Pa*_*i*_:

(1) $$P(X)=P_{G}(X)=\prod_{i=1}^{n}P_{G}\left(X_{i}|Pa_{i}\right)$$

*P*_*G*_(*X*_*i *_| *Pa*_*i*_) is the conditional probability distribution of *X*_*i *_given its parents in *G *or the marginal distribution when *Pa*_*i *_= ∅ . In this study, we use discrete random variables to model the gene expression levels and non-parametric modelling (e.g. Conditional Probability Tables or CPT) to represent the conditional probabilities. CPT present at least two main advantages. They enable representation of any complex regulatory interactions without requiring fixation of the nature of the interactions before learning and they also lead to very simple maximum likelihood estimators. The parametrisation of the BN relies on the coefficients of the CPT : {$\theta_{ik}^{l}$} with *k*, a given state of variable *X*_*i*_, and *l *a given configuration of its parental set *Pa*_*i*_

$$P_{G}(X_{i}=k|Pa_{i}=l)=\{\theta_{ik}^{l}\}$$

In this work, the *Bayes Net Toolbox *(*BNT*) [24] was used in order to create, manipulate and learn BN.

### Structure learning as an optimisation problem

In this part, we present a general and classical approach for BN structure learning often called the *score and search *method.

To identify both structure *G *and parameters given a sample of size *s*, *D *= (*x*^1^,...,*x*^*s*^) of *n *random variables *X *= {*X*_1_,...,*X*_*n*_}, we first need to define a scoring metric that evaluates how the structure and the parameters fit the data. In the case of biological networks, it is not possible to state what is the true cost function at stake. In order to infer the model from data, we know from the learning theory that the scoring metric should incorporate a term responsible for data fitting and a term that controls the complexity of the model.

The Bayesian Information Criterion (BIC) fulfils these requirements and offers a simple way to evaluate a BN structure. BIC was first defined by Schwarz in 1978 as a general proposal for estimating the complexity of a statistical model. Considering G, the set of all possible DAGs containing the aforementioned *n *variables, the best DAG structure $\hat{G}$ can be determined by selecting in G the graph structure *G *that minimizes the BIC:

$$\hat{G}=argmin_{G}\{-2\log P(D|G,\hat{\theta })+K_{G}\log(s)\}$$

where $\hat{\theta }$ is the maximum likelihood estimate of *θ*, the set of parameters of model *G *: $\hat{\theta }$ = *argmax*_*θ*_*P*(*D *| *G*,*θ*) and *K*_*G *_the number of free parameters of model *G*.

For the class of models that we chose and given the i.i.d. data, the likelihood can be expressed as follows

$$P(D|G,\theta )={\prod_{i}\prod_{k}\prod_{l}\theta_{ik}^{l}}^{N_{ik}^{l}}$$

with exponent $N_{ik}^{l}$ being the number of co-occurrences of both *X*_*i *_= *k *and *Pa*(*X*_*i*_) = *l *in the data. Therefore, the BIC can be rewritten as follows:

$$BIC(G)=\sum_{i}\sum_{k}\sum_{l}-2N_{ik}^{l}\log({\hat{\theta }}_{ik}^{l})+K_{G}^{i}\log(s)$$

with $K_{G}^{i}$ the number of parameters in the CPT of *X*_*i*_. ${\hat{\theta }}_{ik}^{l}$ is the maximum likelihood estimate of $\theta_{ik}^{l}$. The P latter can be computed by $N_{ik}^{l}/N_{i}^{l}$ where $N_{i}^{l}=\sum_{k}N_{ik}^{l}$ is the number of times the *X*_*i*_'s parental configuration equals *l *. Since it relies on frequencies, computing these estimators from data is straightforward. This allows to dedicate most of the computational time to the exploration of the structure space. Note that the BIC can be read as the sum of local scores: one local score only depends on the parental set of the node for which it is computed.

To achieve BIC minimization, an appropriate search in the space of candidate graphs has to be defined. To avoid testing all of the possible graphs, searches based on appropriate heuristics are usually preferred. In this work, we use Evolutionary Algorithms to explore the solution space. Various implementations of EA are described as well as their qualitative and quantitative performances assessed on bio-realistic data. Finally, a comparison with classical structure learning algorithms is given. The next section introduces these methods and the results of the testing.

## Results and discussion

### Learning Bayesian network structures with evolutionary algorithms

In this work, we propose to use EA [25,26] to design regulatory network structures such as BN structures. The search for the BN structure, which minimizes the Bayesian Information Criterion (BIC), is implemented through a steady state genetic algorithm whose synopsis can be found below:

• Initialisation : A population of BN structures is randomly generated and then evaluated according to a fitness function

• Repeat until a stopping criterion is satisfied :

1. Pick two genitors at random from the population : P1 and P2

2. Produce a pair of offspring by recombining P1 and P2 : C1 and C2

3. Mutation may be applied to promote diversity : C1 and C2 undergo random edges additions or deletions

4. Evaluate C1 and C2 by computing their fitness function

5. Discard two individuals from the current population to recover the initial population size. To favour the survival of good candidate models, a selection scheme based on the chosen fitness function is used.

#### Structure representation and recombination

In EAs, candidate models are coded with (binary) vectors called chromosomes. Here, their constituents are termed EA-genes. Practically, EA-genes can take on multiple values from any finite alphabet.

Evolving BN structures is a hard task and DAGs encoding turns out to be a crucial issue. Two global strategies can be considered regarding this question: direct and indirect searches.

*Indirect search *[27,28] performed the search in the set of precedence orders of the DAG nodes, when *direct search *works directly in the set of DAGs. A precedence order defines the set of potential parents for each node. For instance, a node *X*_*j *_must precede a node *X*_*i *_in this precedence order to be a parent of *X*_*i *_in the graph. We chose the second alternative and directly evolved BN structures as performed in [29-32]. Since the search is conducted directly over the space of DAG, we faced the classical problem of producing infeasible solutions (digraph with cycle). A first answer is given in [33] to deal with this situation. EA-genes coding elementary edges of both genitors are put together and then injected in the future offspring according to some specific rules in order to maintain feasibility and increase the amount of information in the new Bayesian network [32]. An alternative approach is proposed in [31] by allowing the production of cyclic digraphs and arbitrarily assigning them a low score. In this paper, we considered the acyclicity constraint *a posteriori *using a repair function to remove cycles from new candidate structures (this process is detailed in the *Methods *section). Moreover, we set an upper bound on the number of potential parents per node to 10, which limited the frequency of cycle appearance. Indeed, for each *X*_*i*_, the number of parameters ${\hat{\theta }}_{ik}^{l}$ to estimate and store in the corresponding conditional probability table grows exponentially as a function of the number of parents of *X*_*i*_. For computational convenience, we chose to limit the size of the parental sets. Therefore, offspring produced by recombination had to respect both the acyclicity and the maximum in-degree constraints.

We focused on a generic pair-wise recombination method to perform the search in the space of BN structures. We used uniform crossover exchanging randomly selected EA-genes between two genitor chromosomes. An exchange rate parametrises the proportion of GA-genes that will be passed from one genitor to another during recombination. Since this process is symmetric, the exchange rate ranges between 0 and 0.5. Recombination efficiency depends on its ability to manipulate meaningful information units. We considered two different codings of BN structures making use of different types of EA-genes.

#### Link Recombination

First, we considered a link-chromosome. This is a ternary vector where each EA-gene *φ*_*ij *_(with *i *≠ *j*) can take three values : 0 if there is no edge between *X*_*i *_and *X*_*j*_, 1 if *X*_*i *_→ *X*_*j*_, -1 if *X*_*j *_→ *X*_*i*_. This compact representation relies on meaningful information units which represent elementary interactions between nodes pairs.

#### Parental Recombination

We also considered the parental-chromosome previously used in [31]. A parental-chromosome is composed of a sequence of *n *GA-genes, each of them corresponding to a parental list *Pa*_*j *_(with *j *∈ {1,...,*n*}). The scoring metric we used to evaluate BN structures can be expressed as the summation of local scores assessing the parental lists. Thus, the search for a high fitted BN structure can be achieved by finding proper associations of high scoring parental lists. From a biological point of view, one may assume that *Pa*_*j *_represent sets of regulators acting jointly on the transcriptional activity of gene *X*_*j *_which justifies exchanging it as a whole.

#### Maintaining the diversity in the population of solutions

A fundamental characteristic of EAs is their ability to search the DAG space from multiple points in parallel. However, as the algorithm goes along, the chromosomes of the population may become very similar. Population homogenisation may prevent the crossover operator from exploring new portions of the solution space.

A common approach to fight fast homogenisation is the mutation operator which introduces diversity by means of random edge additions and deletions among candidate models. This allows the algorithm to escape from local minima and exploration of new area in the search space.

Alternatively, niching approaches tend to maintain diversity by limiting the scope of selection processes to subsets of similar individuals. In this study, we used Deterministic Crowding (DC) [34], a crowding method [35] that takes place after reproduction and provides a restricted replacement scheme among similar individuals: each offspring replaces the most similar genitor if it is fitter. Further details regarding Deterministic Crowding are provided in the *Method *section.

Mutation and DC occur at two different steps of the classical process of GA. We will compare results obtained with mutation and/or Deterministic Crowding. When DC is not applied, we used an elitist replacement strategy: offspring replace the two worst individuals of the population if they have higher scores.

#### Benchmark and evaluation metrics

In order to evaluate the performance of a structure learning algorithm, we need to measure its ability to recover the true structure of the regulatory network. Currently there is no gold standard that serves as a benchmark for evaluation, including both real static data and full knowledge of existing pathways. When introducing a new approach, modellers classically use artificial data to test the algorithm in various conditions of size of the simulated samples.

We considered the synthetic model proposed in Le *et al*. [22], which is a bio-realistic Bayesian network based on established knowledge on the insulin regulatory network with a moderate size (35 discrete random variables) and complexity (parsimonious topology). This insulin DAG is the reference graph for the study. We generated samples with various numbers of measures for the 35 genes.

Prior to defining the evaluation metrics that measure the learning performance, it must be noted that BIC is a Markov equivalent scoring metric; therefore, it yields the same score for any DAG belonging to the same equivalence Markov class, i.e. encoding the same statistical model [36]. Formally, an equivalence Markov class is represented by a partially directed acyclic graph (PDAG). It gathers all of the DAGs with the same graph skeleton (undirected structure) and the same set of immoralities [37]. Since the learning process can not discriminate between Markov equivalent DAGs on the basis of only observed data, the analyses were performed on the PDAG containing the best DAG obtained for each run. In the evaluation of the learning process, the best PDAG we found was compared with the PDAG corresponding to the reference model.

As in [22], the metrics used are *sensitivity *($\frac{tp}{tp+fn}$), *specificity *($\frac{tn}{tn+fp}$), and *positive predictive value *($\frac{tp}{tp+fp}$). A true positive (tp) is an edge which appears both in the learnt graph and the reference graph, with the same orientation if it is oriented in the learnt graph. An undirected edge in the learnt graph is considered as a true positive even if it is directed in the reference graph since no speculation is made regarding its orientation. A true negative (tn) is the absence of any edge between two specific nodes in both the learnt and true graphs.

However, the *specificity *did not appear to be relevant. The introduction of an upper bound on the number of parents per node as well as the constraint on the network complexity in the BIC ensured the generation of parsimonious solutions. Since both the learnt and the reference network structures presented a small number of edges, the number of true negatives was always very high in comparison with the number of negatives (*tn *+ *fp*) and therefore the *specificity *was not discriminative.

Finally, we only retained *sensitivity *and *positive predictive value *as quality metrics since the computation duration (up to a few hours on a conventional desktop) will always be short compared to the months which are necessary to achieve a biological experiment. The aim of genetic regulation network learning is to provide clues for gene/protein interaction discovery which have to be biologically validated. Therefore, one has to ensure a high *ppv *to avoid unnecessary costly and time consuming experiments.

In the next section, the performances of the EA optimisation depending on the recombination strategies and diversity preservation methods are first reported. Second, the most promising EA implementation we have found is compared with alternative learning algorithms like greedy search, K2 and MCMC algorithms.

### Exploring the search space with evolutionary algorithms

#### A comparison of various evolutionary approaches

First, we compare the ability of an evolutionary algorithm to recover the true structure of the reference network depending on the recombination strategy it employs and the use of mutation and/or Deterministic Crowding. The results are expressed in terms of *sensitivity *and *ppv *in Table 1. The empirical means and standard deviations of the quality metrics are computed from 10 runs. The 10 runs were performed on 10 different i.i.d. training samples of size 300. The size 300 has been chosen as a balance between the small sample size of real data that we can hope for and the standard in BN learning.

**Table 1:** Comparison of the sensitivity and positive predictive value of various evolutionary strategies

	NoDC/NoMut		NoDC/Mut		DC/NoMut		DC/Mut	
Recombination	SENS.	PPV	SENS.	PPV	SENS.	PPV	SENS.	PPV

Link R. (High Rate)	43 ± 4	61 ± 12	61 ± 6	58 ± 8	63 ± 3	84 ± 5	68 ± 4	74 ± 8
Link R. (Low Rate)	18 ± 7	18 ± 8	42 ± 8	22 ± 8	68 ± 4	82 ± 9	68 ± 4	80 ± 6
Parental R. (High Rate)	23 ± 7	26 ± 8	56 ± 7	38 ± 4	48 ± 3	68 ± 10	66 ± 4	69 ± 6
Parental R. (Low Rate)	12 ± 5	12 ± 5	33 ± 6	14 ± 4	61 ± 4	79 ± 6	60 ± 2	63 ± 7

We report the ability of link and parental recombination to explore the search space for high (0.4) and low (0.1) exchange rates. Results for link-recombination with high and low exchange rates are respectively presented in rows 1 and 2. Results for parental-recombination with high and low exchange rates are respectively shown in rows 3 and 4. In the first column (NoDC/NoMut), we consider the results for these various recombination strategies when neither mutation nor Deterministic Crowding were used. We only use mutation or Deterministic Crowding in column 2 (NoDC/Mut) and column 3 (DC/NoMut), respectively. Mutation and Deterministic Crowding are used jointly in column 4 (DC/Mut). In a given cell, the figures correspond to the results obtained across 10 runs performed on different and independent datasets with a size of 300. These results are presented as the mean ± the standard deviation of the *sensitivity *(SENS.) and *positive predictive value *(*PPV*) across the ten runs. For the sake of clarity, they are expressed as percentages.

The population size was set to 200 DAGs and the stopping criterion of the algorithm is the following: no improvement occurs during at least 1000 iterations.

We report the ability of link and parental recombination to explore the search space for a high (0.4) and a low (0.1) of the exchange rate: rows 1 and 2 are devoted to link recombination results, while rows 3 and 4 present the parental-recombination results. In each table, results are reported in various conditions: neither mutation nor niching (column 1), use of mutation (column 2), use of deterministic crowding (column 3) and use of both (column 4). When mutation is used, it is characterized by a small mutation rate implying about 2 edges modifications per DAG.

Considering the first columns of Table 1, we can see that for both link and parental recombination, the higher the exchange rate, the better the results. This was expected since a higher exchange rate favours a higher mix of EA-genes among candidate models. Thus, the exploration of the search space is accelerated through the generation of a wider variety of candidate models. For a given exchange rate, link-recombination outperforms parental-recombination. It performs recombination at a finer level (elementary interactions) than parental-recombination which, by manipulating large subsets of conditional (in)dependencies, explores the search space more slowly.

Adding the mutation operator (Columns 2) increases the *sensitivity *we obtain with every recombination methods. This improvement is particularly important for the less effective ones. For a low exchange rate, mutation allows attainment of new graph topologies that would not be considered due to the slow mix of EA-genes. For parental-recombination, each EA-gene can take on a huge number of values depending on the large number of parental lists that each node can admit. Since these parental lists are exchanged globally, they will be maintained unchanged during recombination. The mutation operator appears to be necessary to modify them and to explore new configurations of parental lists.

Using Deterministic Crowding (Columns 3) also increases the *sensitivity *of the recombination methods, especially for those with a low exchange rate. However, while the mutation operator fails to improve the *ppv*, Deterministic Crowding significantly increases this quality metric for all recombination methods and parameter values. Finally, the differences we previously observed among them tended to vanish. This improvement comes from the ability of Deterministic Crowding to prevent homogenisation of the population and delay the convergence of the algorithm. This allows even the less effective recombination strategies to pursue searches toward better minima.

Finally, adding the mutation operator to the Deterministic Crowding (Columns 4) leads to balanced results: we observe a moderate *sensitivity *improvement and a loss of *ppv *for the high exchange rate. It must be noted that the various evolutionary approaches we previously compared do not perform the same number of fitness function evaluations until reaching convergence. The biggest difference is related to the use of niching. In our experiments, an EA with link-recombination, a high exchange rate, a small mutation probability and using the Deterministic Crowding performs between 40000 and 50000 function evaluations. The same EA without Deterministic Crowding requires between 20000 and 30000 function evaluations. Considering these values are of the same order, the conditions of a fair comparison are granted for these algorithms.

DC significantly improves the learning process and its performances clearly balance the absence of mutation. Since using both mutation and DC does not yield better overall results than using DC alone, the need for random modification could be discussed. However, for the remaining part of this paper, we chose to use both. DC improvement is obviously of greater benefit for recombination methods which usually fail to efficiently explore the solution space. In order to get a better understanding of the improvement promoted by this niching technique, we propose to study its effect on the homogenisation of the population. For this purpose, we use an adapted visualisation method: the Kernel Principal Component Analysis (KPCA).

#### Visualization of the effect of Deterministic Crowding during learning

The impact of Deterministic Crowding on the exploration of the DAG space can also be evaluated by visualizing how much the DAG structures produced by the two different evolutionary strategies differ. For this purpose, we used Kernel Principal Components Analysis (KPCA) introduced by Schölkopf *et al*. [38] to project DAGs in 2D space. KPCA consists of applying the standard PCA in a feature space endowed with some kernel *k *as an inner product. A kernel *k *is a similarity function that is positive semi-definite. KPCA provides one way to apply PCA to complex objects for which a kernel can be defined. Kernels between graphs were introduced by Gaertner in [39]. Particularly, he proposed the direct product kernel based on the direct product graph.

Given two directed graphs noted *G*_1 _and *G*_2_, we define *E*_× _as the adjacency matrix of their direct product graph *G*_1 _× *G*_2_, given by the element by element product of the adjacency matrices of *G*_1 _and *G*_2_: ∀(*i*,*j*) ∈ {1,...,*m*}^2^, *E*_×_(*i*,*j*) = *A*_1_(*i*,*j*)*A*_2_(*i*,*j*), where *A*_1 _and *A*_2 _are respectively the adjacency matrices of *G*_1 _and *G*_2_.

The direct product kernel *k*_× _is defined by :

$$k_{\times }(G_{1},G_{2})=\sum_{i,j=1}^{m}\sum_{n=0}^{\infty }\frac{\beta^{n}}{n!}E_{\times }^{n}=\sum_{i,j=1}^{m}\exp\beta E_{\times }$$

where *m *is the number of vertices of the graphs, *β *∈ ${ℛ}^{+}$ and *exp *is matrix exponential.

We chose the normalised version of the previous kernel:

$$k_{\times }^{norm}(G_{1},G_{2})=\frac{k_{\times }(G_{1},G_{2})}{\sqrt{k_{\times }(G_{1},G_{1})\cdot k_{\times }(G_{2},G_{2})}}$$

Reducing the visualization in a 2D space implies some loss of information, but it provides an understandable picture and relevant trends of the population behaviour.

During the learning process, the population of BN structures is recorded every 2000 generations. In order to emphasize the evolution of their distribution from one generation to the next, we apply KPCA to the individuals of two consecutive recorded populations (e.g. population at generation 2000 and 4000).

We present the evolution of candidate solutions for EA depending on the application of niching, for link and parental recombination with a high exchange rate. To avoid interferences with niching regarding the control of fast homogenisation, the mutation operator was disabled. For these tests, we used the *SVM and Kernel Methods Matlab Toolbox *[40].

**Figure 1:**
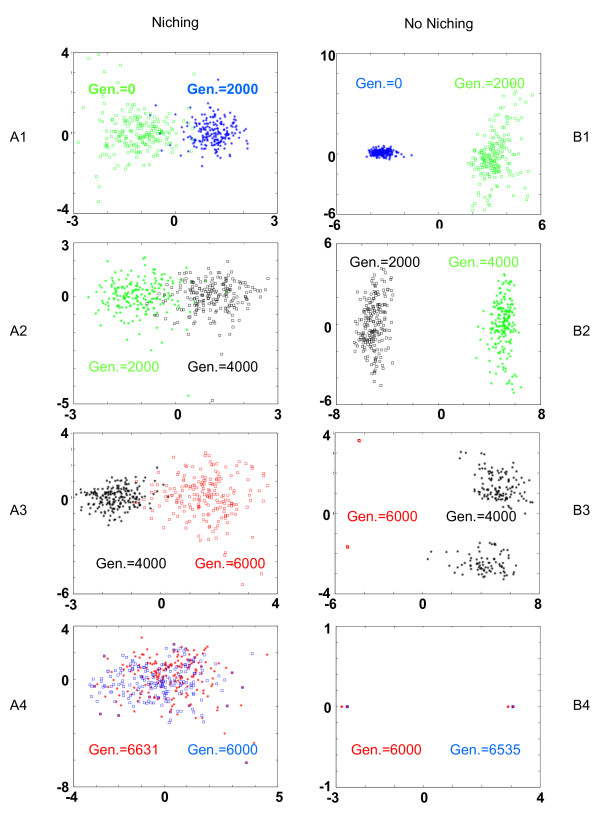
**Evolution of the EA for a genetic algorithm with link-recombination**. These figures show the evolution of the population distribution during the learning process using link-recombination with niching (A1-A4) and without niching (B1-B4). The populations were recorded every 2000 generations and after the algorithm's convergence. Each plot represents the graphs of two populations sampled consecutively. The graphs of both populations are represented as points on a 2D map using *Kernel Principal Composant Analysis*.

**Figure 2:**
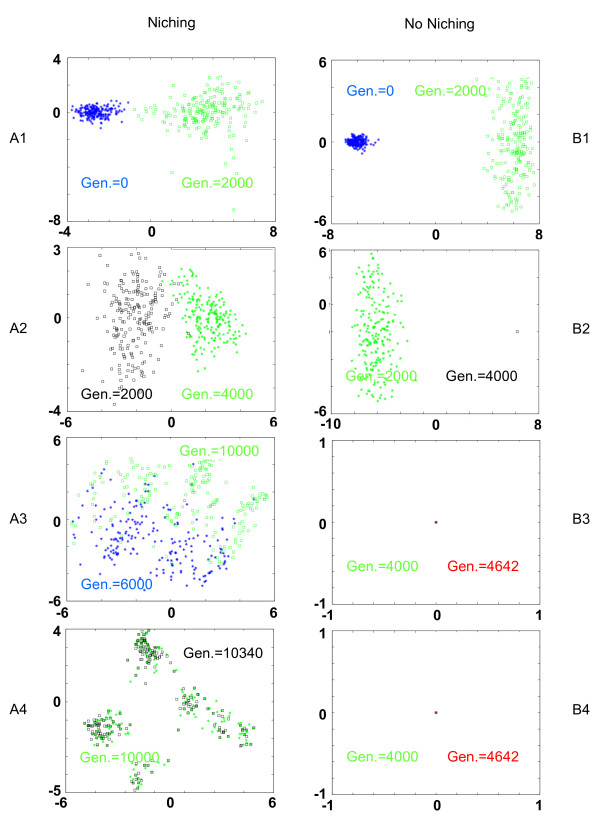
**Evolution of the EA for a genetic algorithm with parental-recombination**. These figures show the evolution of the distribution of the population during the learning process using parental-recombination with niching (A1-A4) and without niching (B1-B4). The populations were recorded every 2000 generations and after the algorithm's convergence. Each plot represents the graphs of two populations sampled consecutively. The graphs of both populations are represented as points on a 2D map using *Kernel Principal Composant Analysis*.

The first and second column of Figure 1 represent the evolution of the population distribution when link-recombination is used with and without niching, respectively. These results are particularly interesting since they closely correlate with the quality of the solutions achieved by the EA. In the absence of niching, the population quickly converges toward a few points (only 4 different solutions were left after 6000 generations), while with niching the population maintained many distinct solutions until convergence. The distributions of the solutions for an EA using parental recombination are depicted in Figure 2 with and without niching in the first and second columns, respectively. In this case, the effect of niching is striking since the population of the EA without niching converges towards a unique point before 4000 generations. Due to niching, the population can preserve a large and heterogeneous population over more than 10000 generations.

Niching postpones population homogenisation and, more interestingly, preserves heterogeneous repartition even after convergence is reached. With such a setup, EA performs a more extensive search of the solution space. At this point, the only question left is the choice of the recombination method. Indeed, if link-recombination with a high exchange rate yields the best results in most cases, Deterministic Crowding seems to reduce most of the differences between the two recombination methods. Therefore, the efficiency of link and parental recombination for a high exchange rate was studied by plotting their learning curves.

#### Introducing the learning curve

The learning curves were built as follows: algorithms were tested for samples with increasing sizes ranging from 50 to 400 cases with a step size of 50. For each sample size, tests were performed on 10 different and independent datasets. We used the same datasets for every algorithm under study. Results are plotted according to the *sensitivity *and the *ppv *metric. Each point corresponding to a given sample size, represents the mean value and the standard deviation for one of these quality measurements across the 10 runs of the algorithms.

The learning curves are given for both parental and link recombination depending on the Crowding implementation. For both recombination strategies, we kept a high exchange rate and mutation was applied.

**Figure 3:**
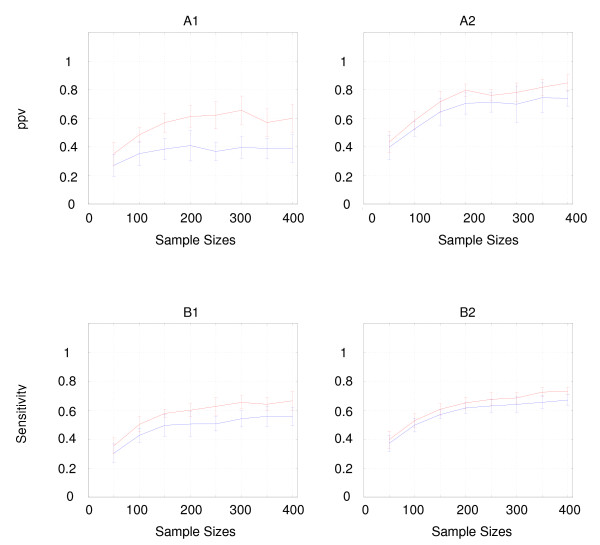
**Comparison of the learning curves of parental-recombination and link-recombination**. For each learning algorithm, the results of the comparison of the graph learnt for various sample sizes and the reference graph are expressed in terms of *positive predictive value *(A1 and A2) and *sensitivity *(B1 and B2). Figures A1 and B1 show the results obtained without niching, while Figures A2 and B2 show the results obtained with niching. The color coding is blue for parental-recombination and red for link-recombination. For each sample size, tests are performed on 10 different and independent datasets. The same datasets are used for every EA. Each point along the curves, which correspond to a given sample size, represents the mean value and the standard deviation of the quality measurement across the 10 runs of the algorithms.

As one can see, for both *ppv *(Figure 3 [A1, A2]) and *sensitivity *(Figure 3 [B1, B2]), link recombination (in red) always performs better than parental-recombination (in blue), even if this difference is much more significant without niching (Figure 3 [A1, B1]) than with niching (Figure 3 [A2, B2]).

Considering these results, we chose link-recombination with a high exchange rate and used both mutation and Deterministic Crowding for the remainder of this paper. The resulting evolutionary algorithm will be compared to other classical structure learning methods for validation.

### Comparison with other structure learning algorithms

#### Introduction to the structure learning algorithms used for comparison

The EA with the setup described above was compared with state of the art and widely used optimisation methods in the field of BN structure learning. The performances of these methods were assessed according to *ppv *and *sensitivity *learning curves which were built as described previously.

First we considered classical *search and score *methods: greedy search and K2 [17] algorithms. The greedy hill-climbing search procedure examines all possible local modifications of the curent DAG, relying on edge addition, deletion, and reversal. In each step it applies the one that leads to the biggest BIC improvement and respects the acyclicity constraint. Like the EA, this algorithm is randomly initialized.

K2 [17] is a deterministic DAG builder. Starting from a graph with no edges, for each vertex, it adds incrementally the parent whose addition most decreases the BIC of the resulting structure. The algorithm stops these additions when adding a single parent do not decrease the BIC anymore. To deal with the acyclicity constraint and reduce drastically the size of the search space, K2 assumes a precedence order on the variables is available. It intends to add as parents only variables which precede the target node in this ordering. As we will see thereafter, this prior information is a crucial advantage.

We also tested a Markov Chain Monte Carlo (MCMC) method [19,22]. Instead of focusing on a single solution optimizing the scoring metric, MCMC samples from the posterior probability distribution of the model *P*(*G*|*D*), which can be approximated with the BIC. Starting from a randomly generated DAG, MCMC constructs a Markov Chain following the Metropolis-Hastings algorithm. After a large number of steps, a state (a DAG in our case) of this chain can be considered as a sample from *P*(*G*|*D*). We performed 44000 steps and only keeps the last 4000 as samples. Each of these DAGs is processed to obtain the corresponding PDAG. These ones are then used to build a single consensus graph by only keeping likely edges that were present in over 50% of them.

Note that the three algorithms we just introduced were set up to respect the constraint on the maximum number of parents per node we imposed to the EA, i.e. parental sets containing more than 10 parents were forbidden.

Finally, we considered full and partial *constraint-based *methods: the Three Phase Dependency Analysis algorithm (TPDA) [41] and the Max-Min Hill Climbing Bayesian network structure learning algorithm (MMHC) [42]. These approaches build the networks by assessing the conditional independencies (CI) among the attribute of the data. In this work, we used a classical hypothesis test to assess the CI : the *χ*^2^-test. We chose the value of 0.05 as a threshold to decide if two variables were conditionally independent.

TPDA is an algorithm that uses an information-theoretic analysis to learn Bayesian network structures from data. It allows for effectively learning a partially directed acyclic graph, requiring only polynomial numbers of conditional independence tests in typical cases.

MMHC relies on a hybrid approach: it combines *constraint-based *and *search and score *technics. MMHC first constructs the skeleton of a Bayesian network using CI tests in a way similar to the famous PC algorithm [43]. It then performs a greedy hill-climbing search to orient the edges. This second phase of the algorithm uses the Bayesian Dirichlet metric [44] with uniform prior to assess the candidate solutions. For these experiments, we used the implementations of K2 and MCMC available in *BNT *[24]. The greedy hill-climbing algorithm came from the *BNT Structure Learning Package *[45]. For the TPDA and MMHC algorithms, we used the implementations proposed in *Causal Explorer *[46].

#### Numerical results

**Figure 4:**
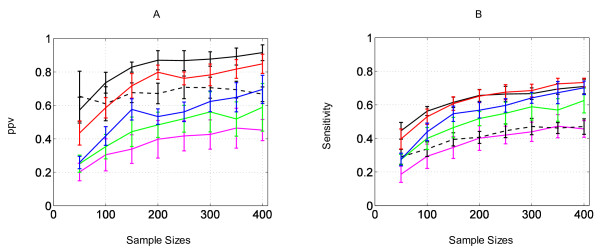
**Comparison of the learning curves of six different structure learning algorithms**. For each learning algorithm, the results of the comparison of the graph learnt for various sample sizes and the reference graph are expressed in terms of *positive predictive value *(A) and *sensitivity *(B). The color coding is green for Greedy search, blue for MCMC, black for K2, dashed black for MMHC, magenta for TPDA, and red for EA. For each sample size, tests are performed on 10 different and independent datasets. The same datasets are used for every algorithm. Each point along the curves, which correspond to a given sample size, presents the mean value and the standard deviation of the quality measurement across the 10 runs of the algorithm.

The plots of the *sensitivity *and *ppv *against the sample size are given in Figure 4 for all algorithms. For both *ppv *and *sensitivity*, the TPDA algorithm yields the worst results. Because of the lack of data, *constraint-based *methods seems to fail in identifying conditional independencies and therefore, in discovering relevant edges. Since it also relies on CI tests for building the skeleton of the network, the MMHC algorithm achieves similar *sensitivity *results to TPDA. However, it shows much better *ppv *results. Indeed, it produces less false positives thanks to the greedy search procedure it uses to perform better edges orientation. Of course, with a finer tuning of the threshold for the CI tests, it should be possible to increase the performances of these algorithms. However, with this setting, they are beaten by *search and score *methods, excepted for MMHC's *ppv *which remains higher.

Since greedy search is a deterministic heuristic which converges towards local optima around the initial point, it usually achieves poor performances. The MCMC algorithm, which relies on an alternative and more efficient strategy, gives better results.

The EA outperforms the MCMC, greedy search and TPDA algorithms for both the *sensitivity *and the *ppv *metrics. The MCMC performance curves depend on the cut-off value used to build the consensus graph. This makes its comparison to other methods difficult. However the large difference between the EA and the MCMC *ppv *curves guarantee that EA performances are better. We note that for small dataset sizes, MMHC remains close to EA in terms of *ppv*.

Finally, K2 is the only algorithm which shows clearly better *ppv *results than EA. This was expected since K2 naturally generates less false positives than any other method. Indeed, prior knowledge is particularly critical when learning from small datasets and, due to node ordering, K2 performs the search in a smaller space including a limited amount of possible edges.

Since the numbers of fitness function evaluations performed by the EA, MCMC, and greedy hill-climbing algorithms are of the same order (tenth of thousands) the comparisons of performances are fair. This question is pointless for K2, TPDA and MMHC which are not stochastic heuristics. Therefore, we cannot consider a restart procedure even if the number of function evaluations performed by K2 and MMHC (during the greedy hill-climbing phase) is significantly smaller.

## Conclusion

In this work, we present an evolutionary approach to undertake the hard task of learning gene regulatory networks from a reasonable amount of observational data. To perform this study, we use synthetic gene expression data sampled from a bio-realistic model of glucose homoeostasis. This model contains a realistic number of nodes and mimics the stochastic behaviour of biological systems. We study the robustness of the learning process with respect to the internal randomness of the EA and the variability of the learning datasets. We study the feasibility of structure learning from reasonably small datasets since gathering even hundreds of microarrays is very difficult.

We first compare various evolutionary strategies in order to find the one which achieves the best structure learning. We show that recombining edges (link-recombination) with a high exchange rate is the best reproduction strategy for the problem at hand. The niching method provides us with the most critical enhancement of the evolutionary approach. We confirm the ability of deterministic crowding to postpone convergence and to preserve diversity in order to allow an extensive search in the space of BN structures. Finally, we validate our evolutionary approach by comparing it with various state of the art learning algorithms. EA outperforms the greedy hill-climbing, MCMC, TPDA, and MMHC algorithms, while K2 yields less false positives than EA. This is expected since K2 is not a fair competitor and only considers a restricted set of potential parents for each node.

Evolutionary learning is a promising framework for the inference of gene regulation networks. An interesting extension may be expected through the study of a more elaborated version of crowding methods, making use of a similarity metric between network structures, such as kernels on graphs. Another perspective of this work is to apply this approach to learn the structure and parameters of dynamical bayesian network [47]. Indeed, as soon as a discrete representation of the dynamic model and a fitness function are available, EA can be applied as we proposed for the static BN.

We illustrate the good performance of EA to infer regulation networks when few learning data are available. With recent developments in EA, like Estimation of Distribution Algorithms that offer means to integrate exogenous information, these algorithms are a very promising way to cope with this very difficult task.

## Methods

### Repair process

Regardless of the type of codings we use to represent candidate solutions as well as the methods we use to manipulate them, recombination always results in two DAGs exchanging a subset of their elementary interactions (edges). After recombination, both genitor solutions present various modifications of their topology which can be expressed through edge additions or deletions. Edge deletions are systematically accepted and applied first to make room in the graph for subsequent additions. Edge additions are rejected if they break either the acyclicity or the maximum in-degree constraints. In this case, we try to add these edges in the opposite direction, by considering both constraints again. We are aware that by reversing the edges we cannot add in the first place, we still biases the recombination process. However, we estimate that simply suppressing them would be even worse, resulting in an important loss of connectivity among candidate BN structures.

To avoid the use of a time consuming cycle detection method, we apply the approach of Giudicci [48]: cycle insertions are detected according to an ancestor matrix which describes the set of predecessors for each vertex: to add an edge from *X*_*i *_to *X*_*j*_, one has to ensure that there is no path from *X*_*j *_to *X*_*i *_. We maintain an ancestor matrix for each DAG in the population. These matrices are updated every time a modification occurs in the corresponding DAG due to the recombination or the mutation.

### Deterministic Crowding

Recombination relies on the exchange of a subset of EA-genes between two genitor chromosomes. Consequently, each offspring inherited the majority of its EA-genes from one of the genitor chromosomes, which will be called the *main contributor*. In addition, only EA-genes coming from the secondary contributor are modified during the repair process. Therefore, we can easily determine which "genitor" is the most similar to a given "child" after recombination, by considering the one from which it inherited the majority of its topological characteristics. Finally, we make the assumption that mutation will not change this similarity. This can be verified by computing the Hamming distance between each child and its genitors. In practice, this appears to be useless since mutation is designed to perform only very few modifications in the offspring (about one or two edge additions or deletions per DAG).

## Authors' contributions

CA implemented the learning algorithms, performed tests and analyzed the results. FA and VF chose the mathematical framework and supervised the work. CA, VF, and FA drafted the manuscript. XG critically reviewed the manuscript. All authors read and approved the final manuscript.
